# Feature-Based vs. Deep-Learning Fusion Methods for the In Vivo Detection of Radiation Dermatitis Using Optical Coherence Tomography, a Feasibility Study

**DOI:** 10.1007/s10278-024-01241-4

**Published:** 2024-09-04

**Authors:** Christos Photiou, Constantina Cloconi, Iosif Strouthos

**Affiliations:** 1 Department of Electrical and Computer Engineering, KIOS Research and Innovation Center of Excellence, University of Cyprus, Nicosia, Cyprus; 2 German Oncology Center (GOC), Limassol, Cyprus

**Keywords:** Acute radiation dermatitis, Optical Coherence Tomography, Feature extraction, Machine learning, Deep learning, Classification

## Abstract

Acute radiation dermatitis (ARD) is a common and distressing issue for cancer patients undergoing radiation therapy, leading to significant morbidity. Despite available treatments, ARD remains a distressing issue, necessitating further research to improve prevention and management strategies. Moreover, the lack of biomarkers for early quantitative assessment of ARD impedes progress in this area. This study aims to investigate the detection of ARD using intensity-based and novel features of Optical Coherence Tomography (OCT) images, combined with machine learning. Imaging sessions were conducted twice weekly on twenty-two patients at six neck locations throughout their radiation treatment, with ARD severity graded by an expert oncologist. We compared a traditional feature-based machine learning technique with a deep learning late-fusion approach to classify normal skin vs. ARD using a dataset of 1487 images. The dataset analysis demonstrates that the deep learning approach outperformed traditional machine learning, achieving an accuracy of 88%. These findings offer a promising foundation for future research aimed at developing a quantitative assessment tool to enhance the management of ARD.

## Introduction

Radiation therapy is routinely used to treat cancer with more than half of all cancer patients receiving some form of radiation treatment during the course of their disease. Unfortunately, radiation can have serious side effects, including acute radiation dermatitis (ARD). ARD, a skin inflammatory condition that develops in reaction to radiation, can be uncomfortable and painful [1–4]. The location of the tumor, the radiation technique, the overall dose, the volume and frequency of radiation, concurrent systemic therapy, concomitant diseases, as well as the patient's individual radiation sensitivity and genetic susceptibility, can all affect the severity of ARD [5, 6]. For patients to benefit fully from radiation therapy, it is crucial to monitor and manage ARD symptoms that include swelling, redness, pain, burning, and itching. Furthermore, pigmentary modifications and loss of hair follicle stem cells are possible. Ulceration and fibrosis could also occur in some unusual cases [7–9]. Despite improvements in radiation therapy configurations and planning strategies, skin toxicity is still a concern, especially for those patients receiving radiation for head and neck cancers [10, 11]. Although the frequency and severity of skin toxicity have decreased over the years, it is still an important clinical challenge for some populations [12].

ARD evaluation and grading, which is usually based on standardized criteria like RTOG and CTCAE, is crucial for clinical therapy. These standards, however, are inherently constrained by subjectivity, unpredictability, and the intricacy of skin reactions. Such visual inspection-based approaches of assessment have significant limitations, for patients with head and neck cancer in particular [13, 14]. First, only descriptive terms describing ARD symptoms are used in the grading guidelines (e.g., the RTOG grading criteria for skin toxicity). Since the rater's experience plays a major role in the visual assessment, there are sometimes large interrater discrepancies. Second, regular ARD evaluation will greatly increase the workload of doctors and will be resource intensive. To address these issues with the present ARD evaluation approach and enhance both ARD management and the overall treatment outcome for patients, it is therefore desirable to develop an automated system for ARD assessment. Assessment accuracy and consistency could be further improved by incorporating machine learning capabilities. By offering standardized, trustworthy criteria, these tools have the potential to lessen subjective diversity among clinicians. Furthermore, research in the management and treatment of ARD could benefit from systems that could enable a more quantitative evaluation and monitoring.

Skin biomarker assessments, thermography, and digital photography, combined with image processing, are among the modalities used to evaluate ARD. While thermography is helpful in identifying inflammation, it is non-specific [15]. Digital photography is constrained by lighting and surface-only views [16]. Skin biomarker assessment, which involves the study of proteins and other molecules, can offer insights into the molecular processes behind dermatitis [17]. Even though machine and deep learning have been used to evaluate skin abnormalities, radiation therapy toxic effects have been relatively less studied. Castanedo et al. presented various data analysis methods for image processing, segmentation, feature extraction, and classification using infrared images that could be used for ARD detection [18]. Another study showed that early thermal markers are predictive of radiation-induced skin toxicity in breast radiotherapy with an accuracy of 87% [19]. Park et al., developed an image-based radiation dermatitis assessment system using a convolutional neural network (CNN) and digital images. The average sensitivity and specificity were 61% and 91%, respectively, in early radiation dermatitis and 78% and 96%, respectively, in severe cases [20]. In addition, using 2263 digital images, another study developed deep CNNs for automatic classification of radiation induced dermatitis. For a two-class problem of normal skin (grade 0) versus dermatitis (grade ≥ 1), the models produced an accuracy of ~ 70%, with sensitivity and specificity of 67–72% and 72–83% [21]. Ruijan et al., demonstrated a deep learning method for automatic assessment of ARD severity in patients with nasopharyngeal carcinoma using 1205 digital images. The overall classification accuracy was 83.0% [16]. It is therefore apparent that further developments are needed both in the hardware, datasets, and algorithms for the detection of ARD. Ideally, the goal should be to predict the appearance of ARD before that even occurs. However, this does not appear to be within the reach of current technologies.

OCΤ is a powerful optical imaging technique that enables non-invasive, real-time, visualization of skin microstructure and microcirculation. OCT can provide high-resolution images, comparable to histopathology (~ 10 μm resolution) in real-time. Furthermore, OCT is non-invasive, enabling imaging of already-injured skin without any contact. Its penetration depth of approximately ~ 2 mm allows visualization of the skin's stratum corneum and epidermis, making it well-suited for ARD diagnosis. In dermatology, OCT has already been investigated for its diagnostic ability, with promising results in the evaluation of basal cell carcinomas, psoriasis, and allergic dermatitis in humans, as well as detecting radiation effects [22–26]. In all these studies, the sensitivity and specificity reached 80% to 96%. However, visual interpretation of OCT images can be challenging, even for skilled evaluators [27]. To overcome this limitation, investigators have attempted to extract various features from OCT images, such as intensity and texture-based features [28, 29], to serve as biomarkers of disease. Recently, novel biomarkers, including group velocity dispersion, scatterer size, and index of refraction, have also been estimated from OCT data and were shown to correlate with malignancy. These new biomarkers reflect both microstructural and biochemical changes of disease and could increase the diagnostic relevance [30–34] of the OCT images. Based on recent experience in the application of OCT in radio-oncology [35–38], establishing diagnostic algorithms that provide objective and actionable management decisions is also essential in order to address the challenges of manually evaluating OCT images. By providing quantitative assessment of skin changes, OCT combined with machine learning, could provide a tool for a more precise and personalized approach to ARD management as well as provide unbiased evaluation of new experimental methods.

In order to determine the feasibility of identifying ARD using OCT, in vivo skin images were acquired from patients undergoing radiation therapy for head and neck cancer. A fully automated algorithm for image segmentation and feature extraction and selection was developed to provide diagnostically relevant information. Traditional (intensity, texture, and fractal) and novel (scatterer size and optical dispersion) imaging biomarkers and machine and deep learning methods were compared for their ability to differentiate normal skin from ARD. This pilot study has produced the first evidence of the potential of in vivo OCT imaging of head and neck ARD in humans with encouraging insights that warrant further investigation.

## Theory and Methods

**Fig. 1:**
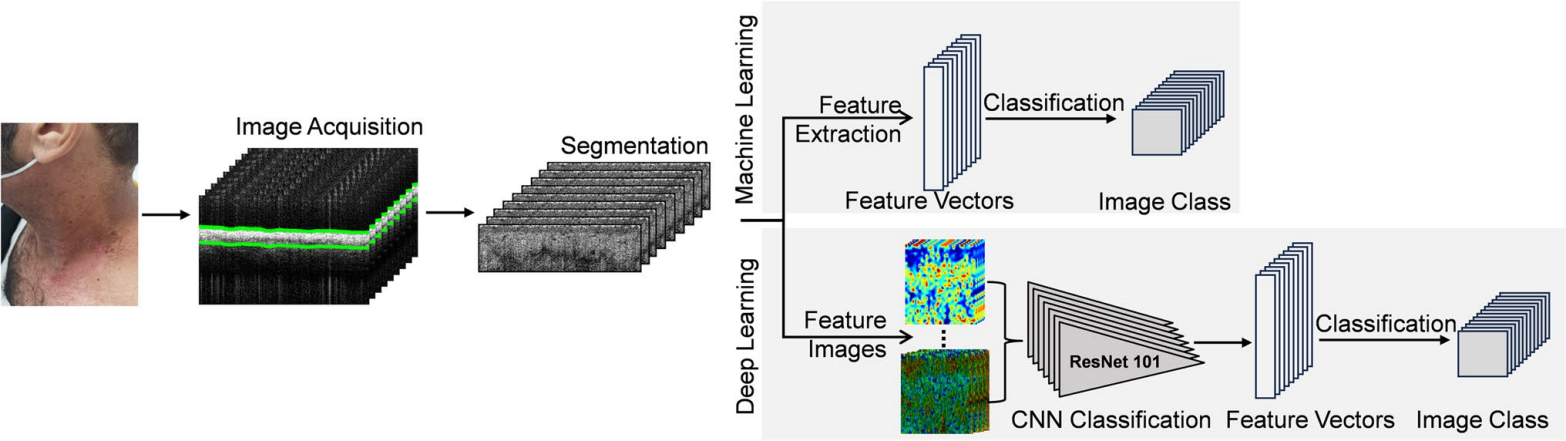
Flow chart of the study showing the two different classification procedures used. After image acquisition and segmentation, feature based ML with feature extraction was applied. Alternatively, feature HSV and pseudocolor images were created, followed by Deep Learning with late fusion

To achieve the goals of this study, image acquisition, preprocessing, feature extraction, and classification with both machine learning and deep learning was performed (Fig. 1).

### Clinical Study and Image Acquisition

**Fig. 2:**
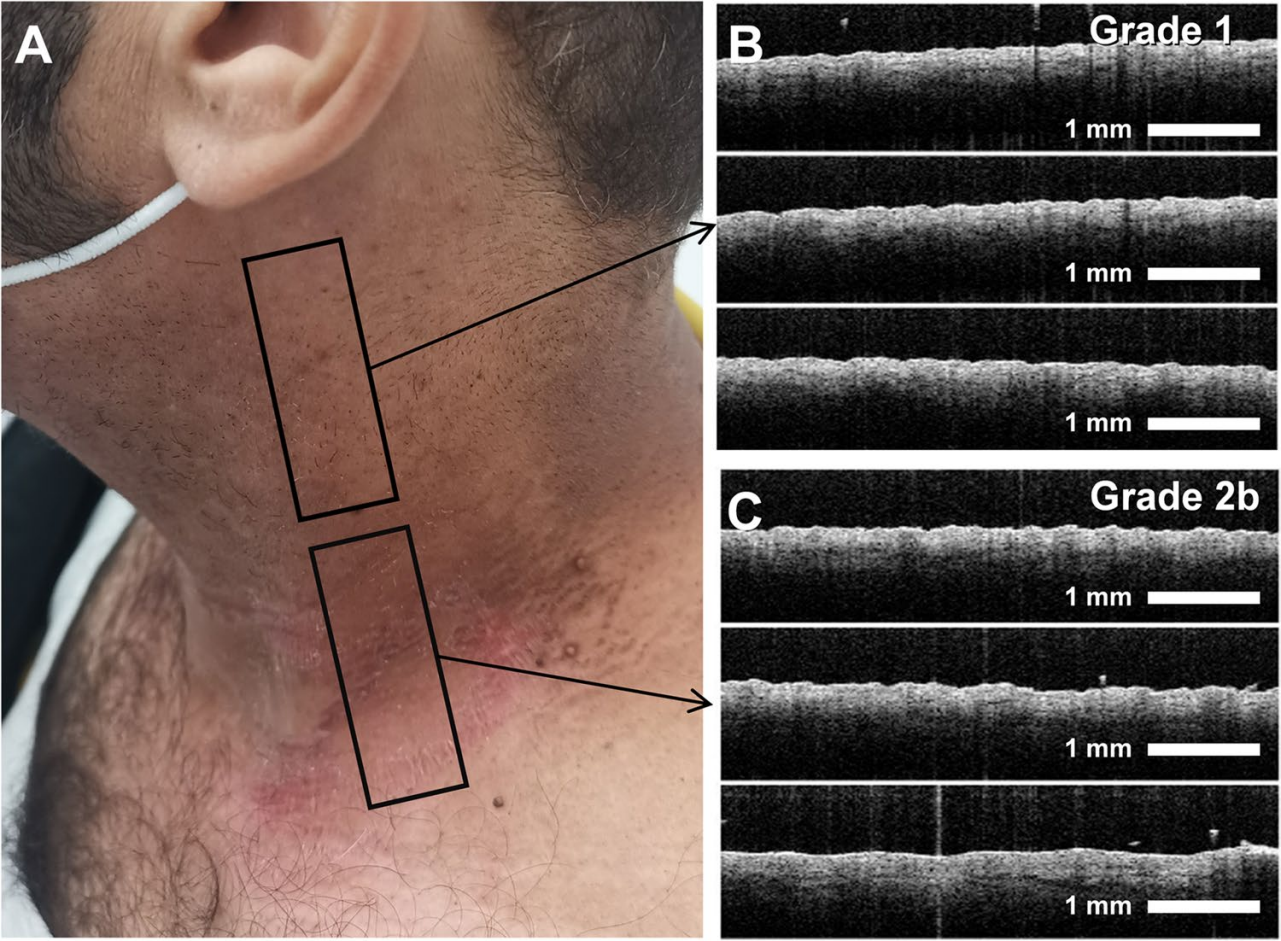
**A** Digital photo of a patient presenting with two different grades of ARD. The top black box encloses an area of Grade 1 ARD whereas the lower black box outlines an area of Grade 2b ARD. **B** Three of the six OCT images acquired from the Grade 1 ARD area. (**C**) Another three OCT images from the Grade 2b ARD area

Twenty-two head and neck cancer patients who were scheduled to receive radiation therapy at the German Oncology Center (GOC) in Limassol, Cyprus, participated in this proof-of-concept trial. The trial has received bioethics approval from the Cyprus National Bioethics Committee. Patients with disabilities, expectant women, those who had recently undergone radiation therapy in the same area, and patients with autoimmune diseases were excluded from the study. All study participants were over 16 years of age. Specifically, one patient was within the range of 30–40 years of age, two were between 40–50 years old, six patients were in the range of 50–60 years, seven fell between 60–70, three between 70–80 age range, and another three were in the 80–90 age range. The mean patient age was 62.3 years with a standard deviation of 12.5. The participants provided the informed consent form prior to the study. After informed consent, the irradiated side of the neck of the subjects was imaged with OCT (Fig. 2). Six images were acquired at 1 cm intervals, covering the region from the mandibular angle to the clavicle. In order to assure proper image spacing, a paper grid guide was used to assist the clinician. Additionally, a photograph of the same area was captured using a digital camera. Imaging was repeated prior to every radiation therapy session, twice per week, until the conclusion of the therapy. The duration of the therapy (6 ± 1 weeks) was determined by the treating physician, resulting in a dataset of 1487 images. Although adjacent images collected from the same patient might be similar, they are not completely redundant since skin microstructure varies from superior to inferior position. Furthermore, the additional images serve as a form of data augmentation to improve the performance of the algorithm. This is further discussed in the results.

During each visit, the patient's ARD grade, at each of the imaging sites, was determined and recorded by the same senior oncologist, with more than eight years of experience. The grade of ARD was determined based on the RTOG scale (grade 0: absence of perceptive lesions, grade I: presence of mild erythema, dry peeling, grade II: presence of moderate to-mild erythema, irregular wet peeling, confined mainly to skin folds and wrinkles, moderate edema, grade III: presence of wet peeling in areas other than skin folds or wrinkles, bleeding induced by minor trauma or abrasion and grade IV: presence of skin necrosis or ulcers) [13, 14]. The images at day 0 (i.e., before the appearance of ARD) were used as the normal dataset of each patient. The imaging was performed with a swept-source OCT system (Santec IVS300), with a center wavelength of 1300 nm, a lateral resolution of 22 μm, an axial resolution of 12 μm in tissue, and an A-scan rate of 40 kHz. Each image consisted of 500 A-Scans, sampling a range of 5 mm. The data were saved in raw interferometric format and subsequently convert to intensity images by FFT and logarithmic processing.

### Image Processing and Feature Extraction

**Fig. 3:**
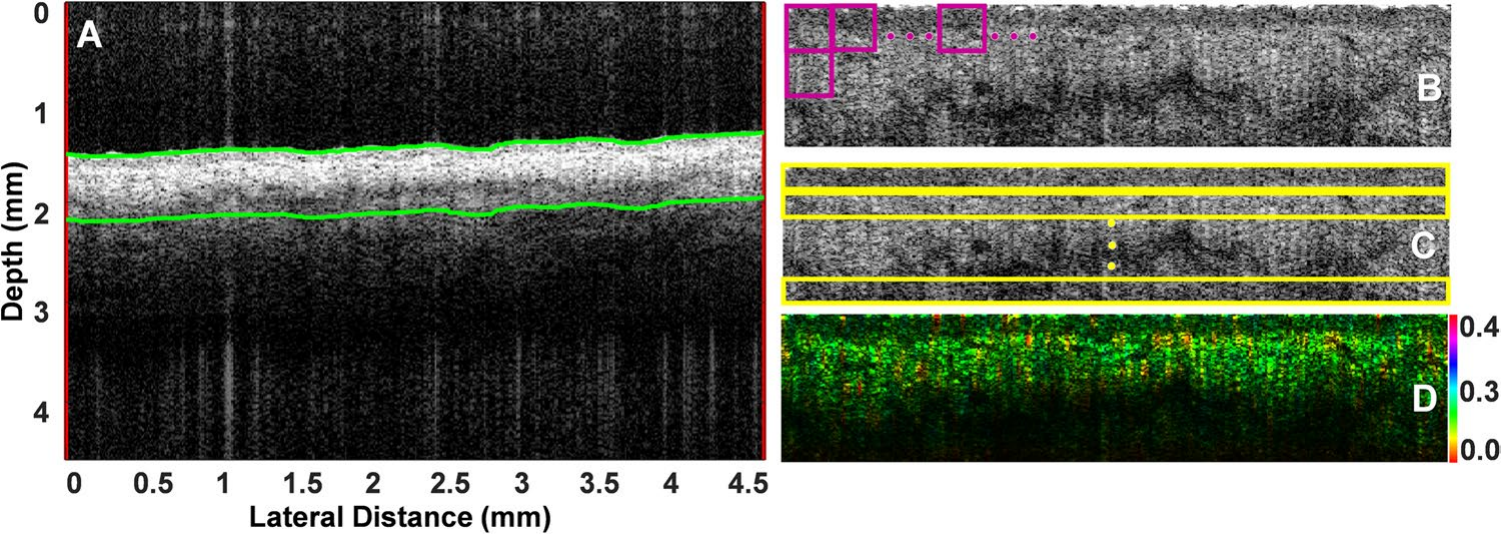
**A** In vivo OCT image of human skin in the neck region. The automated algorithm segmented a given depth of skin (in this example 0.650mm in air (or approximately 0.450 mm in tissue), outlined by the green lines). The vertical red lines mark the portion of the image used (in this case the entire image). **B** The flattened segmented portion which was used to calculate intensity, texture, fractal, and scatterer size features at each distinct-window neighborhood (purple squares). **C** The same image as in (**B**), was used to determine the optical group velocity (GVD) dispersion based on the speckle resolution degradation at progressively increasing depths (yellow rectangles). **D** For each feature, a new image was created where the value of the features (in this example, scatterer size) was overlaid on the intensity as HSV color

An automated algorithm was developed to segment the OCT images of the skin by finding the top surface of each image and subsequently isolating a segment containing the epidermis and part of the dermis (Fig. 3A, between the green lines, and Fig. 3B). The depth was determined by optimizing the classification results. The algorithm used automatic thresholding, with Otsu’s method, and morphological processing of the binary image to determine the borders of the tissue. Morphological processing included image normalization and filtering (median), along with image open and close functions in Matlab to remove redundant and misleading information. Subsequently, several features were extracted either from individual neighborhoods of the image (Fig. 3B, purple squares) or from strips at various depths (Fig. 3C, yellow rectangles). The details regarding each feature are explained below. The value of each feature at each image location was used to create both a pseudo-color image (where each pixel was assigned a color based on the value of the feature) or a colorized image where the value of the feature was overlaid as color over the intensity image (i.e., the hue in a hue-saturation-value (HSV) image, for example Fig. 3D.

Features were extracted from the segmented portion of each image. Various neighborhood sizes were evaluated and the optimal for classification was selected. For each neighborhood in the image, the following features were extracted:

(i) *First order intensity statistics:* Extraction of various first-order intensity statistics, such as the mean, standard deviation, variance, skewness, median, kurtosis, minimum, mode and maximum of the intensity were calculated from each neighborhood of each segmented portion of the images. These features provide valuable textural information. For the purposes of feature-based machine learning, the statistics of each feature over the entire image were also calculated resulting in 90 features per image.
(ii) *Gray Level Co-occurrence Matrix (GLCM):* The Gray Level Co-occurrence Matrix (GLCM), is used to effectively measure perceptual texture image qualities. Four essential characteristics, the correlation, contrast, homogeneity, and energy, can be estimated using this approach. The correlation coefficient gauges how closely two pixels in an image are related to one another. While a low correlation suggests that the two pixels tend to have different values, a high correlation suggests that they are closer together. Contrast quantifies the contrast differences between neighboring pixels. A low contrast implies a small change between neighboring pixel values whereas a high contrast suggests a significant difference. The degree of similarity between adjacent pixels in an image is measured by homogeneity. When homogeneity is high, values of adjacent pixels are comparable. In contrast, when homogeneity is low, values of adjacent pixels are different. Finally, energy quantifies the overall level of uniformity in the neighborhood and indicates a more uniform distribution. Each GLCM feature was extracted at four directions (0, 45, 90 and 135 degrees) and for three different offsets (1, 3, and 5 pixels) from the center of the neighborhood. When the statistics of each were calculated over the entire image, the result was 600 features for feature-based machine learning.
(iii) *Fractal Dimension (FD)*: An effective measure of the complexity and irregularity of image structures is the fractal dimension. It is especially useful in medical imaging due to its capacity of identifying the increased irregularities often associated with disease. The box counting approach is used in this work to calculate the statistics of the FD distribution for all the neighborhoods of each image [29]. For feature-based machine learning, the statistics over the entire image resulted in an additional 90 features.
(iv) *Novel feature – Group Velocity Dispersion (GVD):* Dispersion, an indicator of the wavelength dependency of the index of refraction, has recently been investigated as a useful biomarker of disease. Since changes in tissue dispersion are a result of compositional/biochemical alterations, this metric can be an invaluable complement to the micro-structural information offered by the features above. By examining the speckle patterns in OCT images, the resolution degradation brought about by GVD as a function of depth, can be measured in situ and in vivo [31]. As with the previous features, the calculation of the statistics over the entire image resulted in 10 additional features.
(v) *Novel feature – Scatterer Size (SS)*: In addition to the intensity information, which is the source of the micro-structural images, the OCT interferograms contain spectral information that, most often, remains unused. However, according to the Mie theory of light scattering, oscillatory patterns in the spectrum are related to the size of the scatterer. A novel metric, the bandwidth of the correlation of the derivative (COD) of the OCT spectrum, has been formulated to estimate the mean size of the scatterers, in this case the nuclei of the cells, in vivo [32]. Calculating the statistics of the COD and SS over the entire image resulted in another 20 features for the feature-based machine learning.

### Feature-Based Classification

The images were segmented at a depth of 0.375 mm in air (or approximately 0.260 mm in tissue) since that was empirically shown to provide the most accurate classification results. This is not unexpected since this depth includes the top portion of the epidermis where some ARD early signs (e.g. desquamation) first appear. The segmented image sections were processed to extract the features from each 21 × 21 pixel (0.21 × 0.063 mm) neighborhood of the 1487 images. Given the large number of features (810 in total) and the fact that many of those attributes are correlated, the processes of feature-based machine learning classification began with feature selection. First, the features were ranked in order of significance using a combination of hypothesis t-test and mutual information Maximum Relevance—Minimum Redundancy (MRMR) algorithms. The final number of features used was selected manually to optimize the classification accuracy, in decreasing order of significance. The feature vectors were normalized prior to classification. In addition, to address the issue of an imbalanced dataset, Synthetic Minority Oversampling Technique (SMOTE) was used to increase the minority class by 20% in order to prevent overfitting of the majority class. SMOTE is a data augmentation approach which selects the instances closer to the feature space and creates new samples at points between the existing ones. Various classifiers were evaluated to differentiate normal skin vs. ARD. The classifiers tested included Linear Discriminant Analysis (LDA), with a pseudolinear discriminant type, Support Vector Machine (SVM), with a linear kernel, k-Nearest Neighbor (k-NN), with 11 nearest neighbors, Naïve-Bayes (NB), and Decision Tree (DT) classifiers. The classifiers were compared in terms of accuracy in a leave-one-patient-out (LOPO) cross-validation scheme to ensure unbiased results. Each image was classified using leave-one-patient-out (LOPO) cross-validation, i.e. all the images from all time points of a patient were included in the test set, to avoid training with correlated images.

### Classification Using Multi-feature Deep Learning with Late Fusion (MFDLF)

**Fig. 4:**
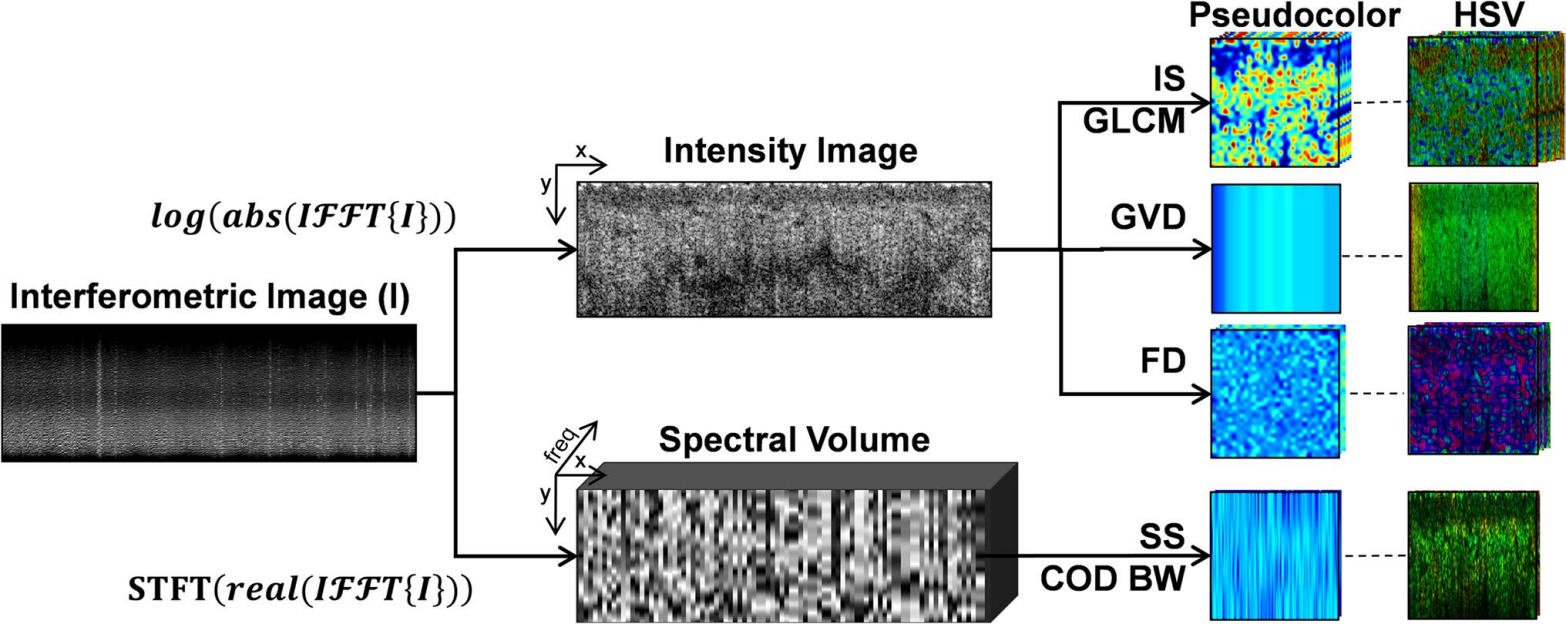
From each OCT imaging location, several feature images are created. First order intensity statistics (IS), GLCM second order statistics, group velocity dispersion (GVD) and fractal dimension (FD) images are created from the intensity image. The spectrally dependent properties of scatterer size (SS) and the bandwidth of the correlation of the derivative (COD BW) are extracted from the spectral content of each raw interferogram

**Fig. 5:**
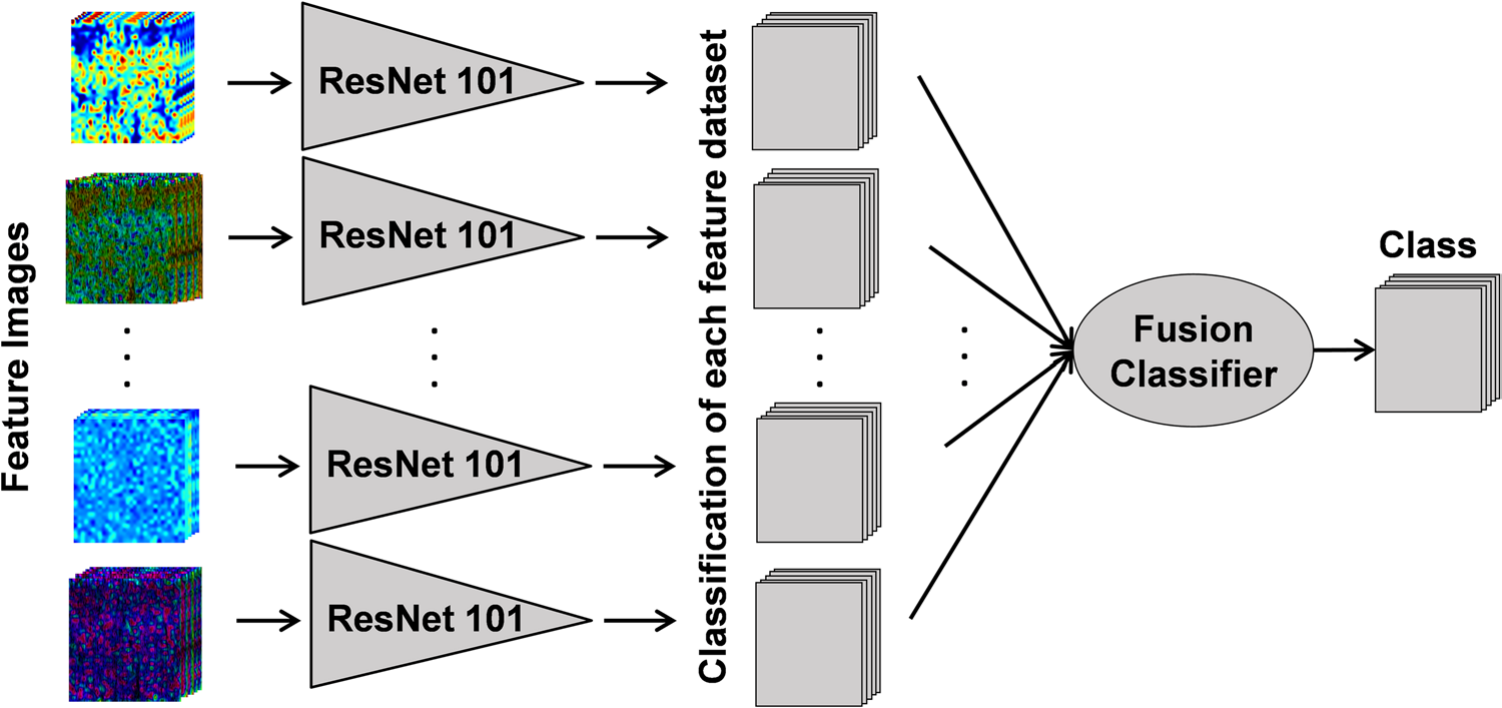
Training flow of the proposed multi-featured deep learning method. Each feature dataset passes through a separate ResNet101 neural network, and the resulting classes are combined into a feature vector which is then passed as the input to a traditional machine learning classifier to produce the final result

A deep learning methodology was also applied to differentiate between normal skin and ARD. Segmentation and feature extraction were performed as described in Sect. 2.3. For the 80 most significant features, based on the feature ranking in 2.3, a pseudo color and an overlay image were created (a total of 237,920 images from the 22 patients) As mentioned above, pseudocolor and HSV images were created for each feature (Fig. 4). These images were rescaled to 227 × 227 × 3 pixels to be adapted to the network architecture. Data augmentation was implemented by applying a set of augmentation operations to the original images during training, generating augmented versions of the images as they pass through the network. The augmentation techniques applied included rotation as well as translation in the x and y scale for each image. A pre-trained ResNet101 neural network was utilized, with the modification of the last layers to incorporate a fully connected layer for two-class classification, followed by a "Softmax" activation, and finally the classification layer. A pre-trained network was selected due to the small number of patients included in this study. The Adam optimizer was utilized with a batch size of 128. The learning rate was set to 0.001 and the network was trained for 35 epochs. The dataset of images corresponding to each feature were classified using a separate network (a total of 160 networks). A new feature vector for each image was created by combining the results of the classification of each of the different feature datasets. Finally, various conventional classifiers were evaluated to combine the results into a final classification of each image (Fig. 5). Each image was classified using leave-one-patient-out (LOPO) cross-validation, i.e. all the images from all time points of a patient were included in the test set, to avoid training with correlated images.

## Results

A Visual examination of the digital photograph and the OCT images of different grades of ARD (Fig. 2), reveals that despite the obvious changes between the two grades in the digital photo, i.e., flaking and redness, distinguishing details that could identify ARD are not easily discernible in the OCT images. This limitation highlights the necessity for computational assistance but also underscores the challenges in detecting ARD from the OCT images.

### Feature-Based Classification Results

The segmented regions from each image were used to extract features for feature-based classification as described above. A window size 21 × 21 pixel (0.21 × 0.063 mm) and a segmentation depth (0.375mm) were selected since they resulted in the best accuracy, after assessing several options (depth up to 0.750 mm and window sizes up to 31 × 31). Fourteen first and second order intensity statistics along with the GVD and SS appeared to be the most significant features resulting in optimal classification. The Linear Discriminant Analysis (LDA) classifier provided the best results for the classification between normal vs ARD classification, with a sensitivity of 86%, specificity of 77% and accuracy of 85% (Table 1). Based on the calculated statistics the LDA classifier provided the best performance, an improvement which was statically significant from the second classifier (SVM) with a p-value of 1.32e-17 [39].

**Table 1:** Classification results for normal skin vs. ARD using feature-based ML

NR VS. ARD	SENSITIVITY [%]	SPECIFICITY [%]	ACCURACY [%]	AUC
LDA	**86.13**	**77.47**	**85.03**	**0.83**
SVM	82.83	64.11	79.92	0.75
NB	79.80	63.13	78.03	0.72
K-NN	73.33	70.10	77.21	0.71
DT	78.37	55.93	76.29	0.67

### Deep Learning Classification Results

As a baseline, the performance of deep learning classification of standard intensity images was evaluated. Using a ResNet101 model the classification accuracy was 70.74% with 83.62% sensitivity and 50.81% specificity. When only three images per patient per session were used, the accuracy was 67.21%. This highlights the importance of augmenting the dataset with additional images. Furthermore, to evaluate the inter-patient variability, the accuracy per patient was considered. In the case of standard intensity images, the individual accuracy varied with a standard deviation ± 10.25%. This is an indication of the extend of inter-patient variability, which implies that an increased dataset could further improve the algorithm’s performance.

For the proposed deep learning method with late fusion, the networks were optimized as described above, resulting in a new feature vector with 160 components for each image. Various combinations of these 160 deep-learning-derived features were evaluated to achieve the highest possible accuracy during the final classification procedure. The optimal choice incorporated 21 of those features, which included the ResNet outputs for the images of five statistics of the fractal dimension, the GVD, as well as sixteen first- and second-order intensity statistics. The list of features, as well as the image format used, is shown in Table 2. A comparison of the results from the various fusion classifiers is shown in Table 3. The Naïve Bayes (NB) classifier, assuming a normal distribution, provided the best discrimination between normal vs. ARD, with a sensitivity of 89%, specificity of 80%, accuracy of 88% and Area Under the Curve (AUC) of 0.85. Based on the calculated statistics the Naïve Bayes (NB) classifier provided the best performance, an improvement which was statically significant from the second classifier (LDA) with a p-value of 4.19e-07 [39].

**Table 2:** Deep-learning-derived features used for the final classification

Deep-Learning Feature derived from	Statistic/Settings	Image Type
**Fractal Dimension (FD) Images**		
FD	Max	Pseudo-color
FD	Mode	HSV
FD	Max	HSV
FD	Skewness	Pseudo-color
**Group Velocity Dispersion (GVD) Images**		
GVD	Mean	HSV
**First-Order Intensity Statistics (IS) Images**		
IS	Skewness	Pseudo-color
IS	Variance	Pseudo-color
**Second-Order Statistics (GLCM) Images**		
Energy	D = 5 (0°)	HSV
Energy	D = 1 (45°)	HSV
Contrast	D = 3 (135°)	Pseudo-color
Average Correlation	D = 1	HSV
Energy	D = 1 (135°)	HSV
Contrast	D = 1 (135°)	HSV
Correlation	D = 1 (135°)	HSV
Average Homogeneity	D = 3	HSV
Homogeneity	D = 5 (135°)	HSV
Contrast	D = 5 (135°)	HSV
Homogeneity	D = 5 (45°)	HSV
Correlation	D = 3 (135°)	HSV
Average Correlation	D = 3	HSV
Correlation	D = 3 (45°)	HSV

**Table 3:** Classification results for normal skin vs. ARD using MFDLF method with different classifiers

NR VS. ARD	SENSITIVITY [%]	SPECIFICITY [%]	ACCURACY [%]	AUC
NB	**88.83**	**80.11**	**88.14**	**0.85**
LDA	86.20	77.32	85.76	0.83
K-NN	90.80	65.42	85.10	0.78
SVM	74.40	69.12	82.02	0.72
DT	75.22	53.32	74.41	0.64

## Conclusions

Radiation induced dermatitis is a common and undesirable side effect of radiation therapy that can severely impair a patient's quality of life. To ensure appropriate and timely intervention, active monitoring and precise assessment of the skin condition are necessary. In this study, traditional and novel imaging features and machine and deep learning methods were compared for their ability to differentiate normal skin from ARD. The results of this in vivo pilot study demonstrate that, despite the limitation of having no visually discernible, distinguishing, microstructural characteristics in the OCT images, carefully selected features and machine learning have the potential to provide accurate classification of ARD. The proposed multi-feature deep learning with late fusion algorithm provided the most accurate results, differentiating normal skin tissue from ARD with an accuracy of 88% (89% sensitivity and 80% specificity). This is an improvement compared to other attempts to automatically detect ARD from photographs of the affected regions.

Although promising, these preliminary results demonstrate that more research is necessary to address the shortcomings of the current study and enhance the algorithms' performance in the use of OCT imaging for ARD diagnosis. One issue with the data generation is that the microscale imaging of OCT makes it exceedingly difficult to precisely localize the scan position. This has minimal effect when skin is normal, therefore relatively uniform, but may affect the images of areas where ARD is not uniform. In a future study, better localization and, perhaps, volume imaging could be used.

One of the main limitations influencing the performance of the proposed method is the small sample size. Data augmentation is a data-space technique to partially address the issue of limited data and, at the same time, balance the data. In the current study, we employed geometric transformations such as rotation and translation for each image. The data imbalance is a result of the fact that only about 20% of patients with radiation-treated head and neck cancer develop severe ARD, out of the 80% to 90% of patients who develop ARD [40]. A clinical study with more participants would be the optimal approach to address these limitations. In addition, more advanced and precise classification techniques can be developed when a larger dataset is gathered. Customized neural networks with earlier and more efficient fusion strategies can be explored, building on the experience of pattern recognition and semantic segmentation of multi-spectral images. Such systems are necessary to both efficiently predict the onset of ARD but also more precisely identify the ARD grade, especially when considering higher grades which may require more urgent medical attention [9].

This study lays the foundation for further investigation in the use of OCT for ARD imaging and may have significant implications for the early diagnosis and management of ARD in patients undergoing radiation therapy. If a tool is successfully developed, it could guide ARD management as well as enable new research that will ultimately improve patient prognosis and quality of life. Furthermore, the application of multi-feature deep learning could be applied to other OCT imaging classification challenges where the microstructure alone does not provide adequate information for an accurate and robust diagnosis.

## Data Availability

The dataset used and analyzed during the current study is available at: https://zenodo.org/record/8238140.
